# Community-based maternal and newborn care: A concept analysis

**DOI:** 10.4102/curationis.v41i1.1922

**Published:** 2018-09-26

**Authors:** Yonas R. Guta, Patrone R. Risenga, Mary M. Moleki, Merertu T. Alemu

**Affiliations:** 1 The World Bank, Ethiopia Country Office, Health, Nutrition and Population (HNP), Ethiopia; 2 Department of Health Studies, University of South Africa, South Africa; 3 Department of Paediatrics and Child Health, Addis Ababa University, Ethiopia

## Abstract

**Background:**

Community-based care can serve as a valuable programme in the provision of essential maternal and newborn care, specifically in communities in low-income countries. However, its application in maternal and newborn care is not clearly documented in relation to the rendering of services by skilled birth attendants.

**Objectives:**

The purpose of the analysis was to clarify the meaning of the concept ‘community-based maternal and newborn care and its relationship to maternal and newborn health’.

**Method:**

Walker and Avant’s and Rodgers and Knafl’s as well as Chin and Kramer’s approaches to concept analysis were followed to analyse community-based maternal and newborn care.

**Results:**

The attributes of community-based care in maternal and newborn health include (1) the provision of home- and/or community-level skilled care, (2) linkages of health services and (3) community participation and mobilisation. These attributes are influenced by antecedents as well as consequences.

**Conclusion:**

The provision of good maternal and newborn care to all clients is a crucial aspect in provision of maternal and newborn services. In order for low-income countries to promote maternal and newborn health, community-based care services are the best option to follow.

## Introduction

Community-based care has become a popular concept in maternal and newborn health care worldwide, but its meaning and effective use, remain debatable because of a lack of clarity on its theoretical and practical meaning (Merzel & D’Afflitti 2003:557). Community-based care can be applied in different health care contexts such as hospital and community settings and in different specialties such as mental health and maternal and newborn care. Lassi, Kumar and Bhutta (2016) describe community-based care as an important component for providing a continuum of care in low-resource communities such as Ethiopia. Their study indicated that the health and well-being of women, newborns and children are inherently linked. Lassi et al. (2016) further confirmed that better health requires that women and children have access to quality services from conception and pregnancy to delivery and during the postnatal period and childhood.

Studies of the South African Medical Research Council (SAMRC) indicate that the concept of community-based maternal and newborn care (CBMNC) is more relevant in relation to community-based care interventions, and these services are important for reducing neonatal mortality rates (SAMRC 2017). Community-based maternal and newborn care is viewed as a good programme for improving newborn survival. However, few studies have analysed the content of care, the cadres, the commodities and the cost-effectiveness of CBMNC (SAMRC 2017). A multi-country analysis of CBMNC study that was conducted in Ethiopia, Ghana, South Africa, Tanzania and Uganda focused on key economic questions relating to the costs of CBMNC programmes, namely their time implications, scale-up implications and budgetary implications. The study found that CBMNC can be delivered for < $1 per person, making it highly cost-effective, even if resulting in an achievable reduction of 1 neonatal death per 1000 live births (< 5% of neonatal mortality) in all the countries assessed (SAMRC 2017).

In Ethiopia, CBMNC is generally provided by skilled birth attendants (SBAs). WHO (2008) defined a skilled attendant as an accredited health professional – such as a midwife, doctor or nurse – who has been educated and trained to proficiency in the skills needed to manage normal (uncomplicated) pregnancies, childbirth and the immediate postnatal period, and in the identification, management and referral of complications in women and newborns. This system has resulted in key challenges regarding the accessibility and availability of SBAs who can manage normal deliveries, recognise and manage obstetric complications or refer patients to the formal health care system (e.g. hospitals or clinics) in time, if necessary (Central Statistical Agency 2012:119, 2014:39).

Skilled birth attendants, as practice-oriented practitioners, should pay considerable attention to developing and clarifying their general knowledge base and their knowledge of CBMNC, in particular. A study conducted by Guta (2016) outlines the common causes of obstetric deaths in Ethiopia, indicating that these causes are, among other things, related to shortages of midwives, poor referral systems at primary health care level coupled with inadequate basic emergency care for both mothers and their newborn babies. This is also aggravated by underfinancing of the services of SBAs. However, other factors leading to greater utilisation of SBAs include cultural norms and societal emotional support to mothers, long distances to health centres and financial barriers (Ministry of Health 2010:38).

The empirical perspectives of Guta’s (2016:193) study laid the foundation for and led to the synthesis and identification of the core theme known as CBMNC. The aim of concept analysis in the study was to explore the meaning of CBMNC in Ethiopia. Guta provided an overview of the method of concept analysis and a description of the results in accordance with the identified categories and the related connotations of CBMNC. Guta formulated a theoretical definition, thus ensuring theoretical validity.

## Methods

The study used the framework described by Walker and Avant (2005:28) and Rodgers and Knafl (2000:78), as well as Chinn and Kramer (2008:192); the researchers explicated the meaning of the concept ‘community-based maternal and newborn care’ to identify the attributes and characteristics of CBMNC and to explore the application of CBMNC in reducing maternal mortality and newborn death. The study examined CBMNC using eight steps, namely selecting a concept, determining the aims or purposes of analysis, identifying all uses of the concept that can be discovered, determining the defining attributes, formulating a model case, identifying antecedents, identifying consequences and defining empirical referents. The study was conducted in Ethiopia and the concept in question was analysed from this context.

### Selecting a concept of interest, relevance, importance and usefulness

Community-based maternal and newborn care was selected as a core concept in this study because it was found by Guta (2016:178) to be the central idea or theme, and all the other ideas are related to it. According to Sengane’s (2013) study, mothers expect midwives to give them moral support and comfort. Hence, a greater understanding of the concept of CBMNC may assist in identifying care needed and care provided by SBAs in communities. Walker and Avant (1995:40) identified three critical questions to be used in concept selection, namely questions of fact, value and concept. This article focuses on the question of concept in order to identify the implications of the identified concept, namely community-based care in the maternal and newborn context.

### Aims of concept analysis

The aim of concept analysis is to distinguish between the ordinary and the scientific utilisation of CBMNC, which is necessary for concept clarification in order to add to existing theory and to develop an operational definition (Walker & Avant 1995:40). Wilson (1963:33) recommends that an analyst determines who might use the concept, and when, how and why it may be used, as a way of identifying the contexts within which the concept is used. ‘Community-based care’ has different interpretations in different contexts, both globally and nationally. The concept ‘community-based care’ was created by Guta (2016:178) to clarify and to describe the meaning of CBMNC within the context of preventing maternal mortality and newborn death in Ethiopia. This context aided the development of a theoretical definition of the concept and guided the meaning of the concept by clarifying the basic elements, structure and functions of the concept (Chinn & Kramer 2008:192; Walker & Avant 2005:66).

### Ethical considerations

Ethical clearance to perform the study was obtained from the University of South Africa (Reference no. REC-012714-039) and the Director of Medical Services of the Ethiopian government, Ministry of Health (MOH) (Reference no. 3.10/072/2015).

## Definitions of concepts

The term ‘community-based care’ has a wide range of meanings. *Stedman’s medical dictionary for the health professions and nursing* (2012:112) defines ‘community-based care’ as the provision of skilled therapy services within a client’s own home or community, with the requirement that the practitioner take into consideration the lifestyle of the client and the cultural and social characteristics of the client’s community.

This study focuses on four models of implicit constructions of community-based care: community as setting, community as target, community as resource and community as agent. Community-based maternal and newborn care is also integrated in this description as the main concept of the study (Steckler et al. 1993:1–153).

The first model (i.e. *community as setting*) was identified by Merzel and D’Afflitti (2003:557–574). According to this model, the term ‘community-based care’ often refers to the community as the setting for interventions. In maternal and newborn care, the setting plays a crucial role too. There is a need to understand the population that needs to change in community-based care as a setting in order to reduce maternal and newborn deaths by using SBAs who are well trained and who understand maternal and newborn care services in the communities. This community-based care consists of assessing, planning and managing maternal and newborn care services as well as referral systems in respect of complications that arise during the provision of these services. Community-based care will help SBAs to identify complications early, ensuring that mothers and babies are cared for properly within their community. Therefore, community-based care as a setting is applicable in maternal and newborn care.

According to the second model (i.e. *community as target*), the term ‘community-based care’ is defined in relation to the community that serves as the target of change. Community indicator projects use data as a catalytic tool to go beyond the use of individual behaviours as primary outcomes (Coulton 1995:173). In the case of maternal and newborn health, changes can be initiated positively within the community in order to reduce maternal and neonatal mortality, which is escalating in Ethiopia.

A third model of community-based care is *community as resource*. The model is mostly used in community-based health promotion services in order to enhance community ownership and participation as essential elements for sustained success in population health outcomes example healthy city projects used in several countries (Duhl & Lee 2000:107–295). In maternal and newborn care, this model can be applied by providing community members, SBAs and pregnant women or women who have just given birth with information regarding maternal and newborn services available in their communities. The study conducted by Mnisi, Peu and Meyer (2012) revealed the importance of training community members in order to prevent health challenges in the community, which is in line with this model. Therefore, in analysing CBMNC this model is also perfect because community is used as a resource for providing maternal and newborn care services in order to improve and promote the health of the inhabitants in those communities.

The fourth model of community-based care is *community as agent*. Although closely linked to the model just described, the emphasis in this model is on respecting and reinforcing the natural adaptive, supportive and developmental capacities of communities. According to Steckler et al. (1993:1–153), communities provide resources for meeting community members’ day-to-day needs.

## Determining the defining attributes of community-based maternal and newborn care

Walker and Avant (2005:68) regard the step of determining the defining attributes or defining characteristics of a concept as the ‘heart of concept analysis’. Once researchers have identified all the different usages of a concept in various fields, the next step is to find characteristics that appear repeatedly. The result of this activity is ‘a cluster of attributes that are the most frequently associated with the concept’ (Walker & Avant 2005:68). This is a process of semasiological analysis, and ‘concept’ actually means an expression and its different meanings.

In this study, determining the defining attributes of the concept of CBMNC was done by making notes on the characteristics of the concept that appeared repeatedly. Three essential and defining attributes have been identified in relation to the concept ‘community-based maternal and newborn care’:

- *Provision of home- and/or community-level skilled care:* Interventions are available where and when they are most needed to significantly save lives in that maternal and newborn care is provided in communities using available resources, which is cost-effective. The model case presented in this article signifies how CBMNC is conducted in Ethiopia. These services are provided by SBAs and the communities benefit from the services provided.
- *Linkages of health services:* Some obstetric and neonatal complications cannot be prevented or treated only at home, and these instances account for 10% of cases in Ethiopia (CSA 2012:126, 2014:45). Women and their newborns must receive lifesaving care as soon as complications happen, during referral and at appropriately equipped health facilities. These connections must be established, maintained, evaluated and redesigned over time as the situation changes. Community-based care allows maternal and newborn care to connect with SBAs and other persons in their community who are responsible for rendering such care. Community-based care in the context of preventing maternal and newborn mortality is, therefore, defined as home- and/or community-level skilled care provided by the community, for the community, with linkages to health facilities to avoid all forms of delay.
- *Community participation and mobilisation:* Community members assess their own health needs and develop and monitor their own solutions to identified problems. Community-based care normally coincides with a high degree of community participation because the onus is on communities to care for their own in addressing the problems at hand. This is also applicable in respect of maternal and newborn care services, where community members can be involved in helping each other regarding these services and identifying SBAs in their communities. Different cases are presented below to reflect the presence of CBMNC in Ethiopia.

## Case description and analysis of community-based care in maternal and newborn health

Walker and Avant (1995:42) advise concept analysts to develop one or more model cases that represent a real-life example of ‘the use of [*a*] concept that includes all the critical attributes of the concept’. In addition, as a next step, Walker and Avant include in their model an examination of additional cases. The inclusion of additional cases originates from Wilson’s model (1963:28–32) and these cases are divided into borderline, related, contrary, invented and illegitimate cases. Walker and Avant point out that an ‘analysis cannot be completed until there are no overlapping attributes and no contradictions between the defining attributes and the model case’. The purpose of this approach is to determine what counts and what does not count as a defining attribute and which characteristics or attributes best fit the concept of interest, thus making the model case stronger. However, all these additional cases are not necessarily included in individual concept studies.

In order to further elaborate on the concept of CBMNC, three categories of cases are provided. The identified cases in this analysis are derived from the findings of the empirical phase of the study, actual examples taken from the researchers’ many years of practical work experience with maternal and newborn care programs and, of course, real-life case models documented in the literature (Mannah et al. 2014:279). The initials that are used to identify each case are fictitious and are not based on the study participants’ actual names. The model case demonstrates all the defining attributes of the concept, while the borderline case contains most but not all of them. This helps to more fully articulate the meaning of the concept. The final, contrary case is a clear example of what the concept is not (Walker & Avant 2005:68).

### Model case and analysis

Few examples identified during the study were highlighted underneath. An old retired midwife R.S., 62 years of age, worked in a district hospital in Ethiopia’s western province for more than 30 years. Following her retirement, she moved back to her remote community and the community chose her as their maternal and newborn health worker through open community involvement.

The retired midwife carried delivery kits with her and assessed pregnant women. If she saw that there were no complications, she conducted deliveries at women’s homes. When she worked, traditional birth attendants (TBAs) and relatives of the pregnant women supported her. She ensured that the TBAs received payment in kind for assisting her with deliveries. Sister R.S. and her community worked in collaboration with the government’s health facilities and offices, reporting relevant matters to them. She provided them with the *kebele’s* (residential site) maternal and newborn care statistics every month. The health facilities, in turn, gave her the contact numbers of key health personnel and ambulance drivers for easy access during referrals. When R.S. referred women, she gave them a referral form. The health facilities also gave her feedback on the women she referred to them. R.S. knew that for most pregnant women, reaching the health facilities, delivering their babies and going back home were not easy in this remote community, and there were usually significant delays. R.S. also knew that the community only supported births at home, especially in the case of first births, and attached a lot of significance to where and how placentas were disposed of. Because some women felt that delivering their babies in the village offered them some privacy, they liked having somebody like R.S. deliver their babies in their homes. R.S. gradually established a ‘wired mothers’ set-up by writing her phone number on pregnant women’s antenatal care (ANC) cards, distributing it at health posts and posting it on health centres’ doors. The midwife was well known by community members. She could usually be called for a home delivery at any time and in the 5 years, as she had started providing community-based MNC services in that community, there were almost no maternal mortalities or newborn deaths reported.

This model case fully demonstrates all three attributes of community-based care, namely the provision of home- and/or community-level skilled care, linkages of households, communities and facilities, and community participation and mobilisation. Although community-based care may seem to be the most appropriate model, there are people who wish to anchor it with clinic-based, hospital-based or some other form of facility-based care.

### Borderline case and analysis

T.U. is a 28-year-old health extension worker in a remote *kebele* of a district health post in Ethiopia’s western province, where she was born and raised. The Ethiopian government developed the Health Extension Programme (HEP) when it realised that basic health services, in general, and maternal and newborn care, in particular, were not reaching people at grassroots level as originally envisioned in the health sector development programme (HSDP).

T.U.’s efforts are met with a positive response by the community and are supported by the government. The community clearly understands her messages about the advantages of making use of the free maternal and newborn care provided at all health facilities.

### Contrary case and analysis

V.W. is a 58-year-old TBA in a remote western province district of Ethiopia where she has spent all her life. V.W obtained traditional wisdom from her grandmother and has been helping women give birth. V.W. has never been trained. In V.W’s community, pregnancy and the events surrounding it are generally viewed as female issues, and the outcome and the *sequelae* (pathological condition) of pregnancies in her community are entirely left to her. The government has neither trained nor supported her and thinks that training TBAs is a waste of resources. Assisting with births is V.W.’s life and the basis of her livelihood. For V.W., life or death is all about luck and is out of her control. She always prays before she assists with a delivery and she is greatly respected and valued. This case reflects an absence of the attributes of community-based care.

## Antecedents of community-based maternal and newborn care

Examination of the antecedents and the consequences of community-based care in averting maternal mortality and neonatal death allows further refinement of the critical attributes, thus facilitating the formulation of the criteria for CBMNC.

Antecedents are those events or incidents that should occur prior to the occurrence of a concept. Antecedents assisted the researchers in identifying underlying assumptions about the concept ‘community-based maternal and newborn care’ (Chinn & Kramer 2008:195; Walker & Avant 2005:73). In this study, the researchers identified the following antecedents:

### Deployment of skilled birth attendants in their own community

Skilled birth attendants are not just skilled but also best suited to working at community level because they understand the local customs in their community. Recruitment processes for this category are the mandate of that specific community. Relevant contact details of SBAs are disseminated to community members in need of their services. The SBAs must be accessible to their clients in order to reduce the need for clients to use taxis, minibuses or motorbikes or bicycles.

### Linkages of skilled birth attendants with health facilities

Community SBAs should be connected to health facilities, which can give them support and supply them with certain basic commodities and consumables. Health facilities can also assist SBAs in autoclaving equipment and instruments. Skilled birth attendants should be given contact numbers of key health personnel and ambulance drivers for easy access during referrals. In addition, health facilities should involve SBAs in certain district health activities. Governmental and non-governmental organisations should also support SBAs by providing them with an initial supply of delivery kits, basic supplies and family planning commodities. If the community has to pay for such supplies, they should be provided to SBAs at subsidised or discount prices. This might heighten SBAs’ motivation and dedication in doing the work properly.

### Regular reporting and feedback

Skilled birth attendants should work in collaboration with government health facilities and send monthly reports on their activities to the district health team. This will help them to get feedback from the health facilities about referrals made and to provide a continuum of care following patients’ discharge.

## Consequences of community-based care

Consequences are those events or incidents that occur because of the occurrence of a concept and that often stimulate new ideas or avenues for research pertaining to certain concepts. These are the outcomes of a concept. Consequences assist researchers in determining often-neglected ideas, variables or relationships that might yield fruitful new research directions (Chinn & Kramer 2008:195; Walker & Avant 2005:73). Possible consequences of the concept of CBMNC are as follows:

### The number of deliveries assisted by skilled birth attendants will increase

Once SBAs have been given in-service education based on their needs and connected to health facilities in their area, the number of deliveries they attend to can be documented. When communities are made aware of the knowledge and the skills that SBAs have acquired, the number of deliveries they assist with will increase.

### Teamwork will be promoted

Teamwork has been described as a dynamic process and an action that involves two or more participants or health care professionals with complementary backgrounds and skills who share common health goals and exercise concerted physical and mental efforts in assessing, planning, implementing and evaluating patient care (Stone & Bailey 2007:259). Gaudes et al. (2007:84) argue that effective team members are able to work interdependently, supporting each other, displaying group cohesiveness, group reliance, respect and a trusting relationship, and sharing the responsibility for their outcomes. Skilled birth attendants sometimes called community midwives by community members, health extension workers, family members and TBAs will work as a team, having regular meetings to discuss achievements and challenges. Skilled birth attendants will focus on safe childbirth, sharing certain tasks such as antenatal care, postnatal care and community mobilisation with other health workers. In well-established community-based care, everyone will work as a team, displaying teamwork skills, which include the ability to resolve team conflicts and to give effective group performance (Stone & Bailey 2007:258).

### The community will be empowered

According to the *Oxford Mini Dictionary and Thesaurus* (2008:220), ‘empower’ means ‘authorise’, ‘enable’, ‘allow’, ‘permit’, ‘license’ or ‘qualify’. Once a CBMNC system has been established, TBAs and family members of pregnant women will gain new knowledge and skills on how to care for pregnant women during the antenatal, the intrapartum and the postnatal periods. Consequently, they will gain confidence in working with registered midwives. This may help community midwives to gain new knowledge on how to provide culturally congruent care and they will experience job satisfaction, resulting in the provision of culturally sensitive care (Luczyriski, Glowrisak-Olszewska & Bossowski 2016:567).

## Defining empirical referents

This serves as the last step of concept analysis. Identifying and defining empirical referents helped the researchers to understand the importance of community-based care in maternal and newborn care as well as its theoretical base as a concept. This contributed to both the content and the construct validity of the model for CBMNC. In the empirical phase of this study, the empirical referents were identified from the health care experiences of pregnant women, women who had given birth and their newborn children, family members of these women, SBAs and different levels of health workers as regards maternal and newborn care practices in the community. Furthermore, the details of empirical referents for categories of the related phenomena were identified.

## Conclusion

The study shed more light on the meaning of the concept of community-based care. This analysis led to the development of antecedents, attributes, model cases and consequences of the concept. Lastly, empirical referents were also highlighted and discussed. The study can provide a basis for the application of the concept of community-based care in the maternal and newborn context in communities. The relevant policymakers can use this analysis to improve maternal and newborn health in low-income areas, which may lead to a decrease in maternal and neonatal complications. Skilled birth attendants’ knowledge can be improved through in-service training, enabling them to identify maternal and neonatal complications early. This may lead to improved referrals by SBAs to health facilities, resulting in a decrease in complications.

## References

[CIT0001] Central Statistical Agency (CSA) [Ethiopia], 2012, *Ethiopia Demographic and Health Survey 2011*, CSA, Addis Ababa, Ethiopia.

[CIT0002] Central Statistical Agency (CSA) [Ethiopia], 2014, *Ethiopia Mini Demographic and Health Survey 2014*, CSA, Addis Ababa, Ethiopia.

[CIT0003] Chinn P.L. & Kramer M.K., 2008, *Integrated knowledge development in nursing*, 7th edn., Elsevier-Mosby, St. Louis, MO.

[CIT0004] Coulton C., 1995, ‘Using community-level indicators of children’s well-being in comprehensive community initiatives’, in Connell J., Kubisch A. & Schorr L. (eds.), *New approaches to evaluating initiatives*, pp. 173–200, The Aspen Institute, Washington, DC.

[CIT0005] Duhl L.J. & Lee P.R., 2000, ‘Focus on healthy communities [theme issue]’, *Public Health Report* 115, 107–295.

[CIT0006] Gaudes A., Hamilton-Bogart B., Marsh S. & Robinson H., 2007, ‘A framework for constructing effective virtual teams’, *The Journal of E-Working* 1, 83–97.

[CIT0007] Guta Y.R., 2016, *Post-mortem lessons: A community based model for averting maternal mortality and newborn death in Ethiopia*, UNISA Suppository, Pretoria.

[CIT0008] Lassi Z.S., Kumar R. & Bhutta Z.A., 2016, ‘Chapter 14: Community-based care to improve maternal, newborn, and child health’, in Black R.E., Laxminarayan R., Temmerman M. & Walker N. (eds.), *Reproductive, maternal, newborn, and child health: Disease control priorities*, The International Bank for Reconstruction and Development/The World Bank, Washington, DC, viewed 05 April 2016, from https://www.ncbi.nlm.nih.gov/books/NBK361898/doi:10.1596/978-1-4648-0348-2_ch14 27227219

[CIT0009] Luczyriski W., Glowrisak-Olszewska B. & Bossowski A., 2016, ‘Empowerment in the treatment of diabetes and obesity’, *Clinical Diabetes* 2016, 5671492, 9 p. 10.1155/2016/567–1492 PMC5206444 28090541

[CIT0010] Mannah M.T., Warren C., Kuria S. & Adegoke A.A., 2014, ‘Opportunities and challenges in implementing community based skilled birth attendance strategy in Kenya’, *BMC Pregnancy and Childbirth* 14, 279 10.1186/1471-2393-14-279 25128479 PMC4262243

[CIT0011] Merzel C. & D’Afflitti J., 2003, ‘Reconsidering community-based health promotion: Promise, performance, and potential’, *American Journal of Public Health* 93, 557–574. 10.2105/AJPH.93.4.557 12660197 PMC1447790

[CIT0012] Ministry of Health (MoH) [Ethiopia], 2010, *Health Sector Development Program (HSDP) synthesis report of 2005/2006–2008/2009*, MoH, Addis Ababa, Ethiopia.

[CIT0013] Mnisi S.D., Peu M.D. & Meyer S.M., 2012, ‘Role of community nurses in the prevention of tuberculosis in the Tshwane Health District of Gauteng’, *Curationis* 35(1), Art. #47, 1–9. 10.4102/curationis.v35i1.47 23327774

[CIT0014] Oxford Mini Dictionary and Thesaurus, 2008, Sv ‘empower’, Oxford University Press, Academic, Oxford.

[CIT0015] Rodgers B.L. & Knafl K.A., 2000, *Concept development in nursing*, 2nd edn., Saunders, Philadelphia, PA.

[CIT0016] Sengane M., 2013, ‘Mothers’ expectations of midwives’ care during labour in a public hospital in Gauteng’, *Curationis* 36(1), Art. #320, 1–9. 10.4102/curationis.v36i1.320 26697615

[CIT0017] South African Medical Research Council (SAMRC), 2017, *Press release*, viewed 12 February 2018, from http://www.mrc.ac.za/Media/2017/37press2017.htm

[CIT0018] Steckler A., Israel B., Dawson L. & Eng E., 1993, ‘Community health development: An overview of the works of Guy W. Steuart’, *Health Education Quarterly Supplement* 1, S3–S20. 10.1177/10901981930200S102 8354649

[CIT0019] *Stedman’s Medical Dictionary for the Health Professions and Nursing*, 2012, Illustrated 7th edn., Sv ‘community-based care/practice’, Medical Group, Stedman’s, North Carolina, USA.

[CIT0020] Stone R.W. & Bailey J.J., 2007, ‘Team conflict self-efficacy and outcome expectancy of business students’, *Journal of Education for Business* 82, 247–263. 10.3200/JOEB.82.5.258-266

[CIT0021] Walker L.O. & Avant K.S., 1995, *Strategies for theory construction in nursing*, 3rd edn., Appleton & Lange, Norwalk, CT.

[CIT0022] Walker L.O. & Avant K.S., 2005, *Strategies for theory construction in nursing*, 4th edn., Pearson Prentice Hall, Upper Saddle River, NJ.

[CIT0023] World Health Organization, 2008, *Proportion of births attended by a skilled health worker updates*, WHO Publication, Geneva.

[CIT0024] Wilson J., 1963, *Thinking with concepts*, Cambridge University Press, Cambridge.

